# Heat Resistance Mediated by a New Plasmid Encoded Clp ATPase, ClpK, as a Possible Novel Mechanism for Nosocomial Persistence of *Klebsiella pneumoniae*

**DOI:** 10.1371/journal.pone.0015467

**Published:** 2010-11-09

**Authors:** Martin Saxtorph Bojer, Carsten Struve, Hanne Ingmer, Dennis Schrøder Hansen, Karen Angeliki Krogfelt

**Affiliations:** 1 Department of Microbiological Surveillance and Research, Statens Serum Institut, Copenhagen, Denmark; 2 Department of Veterinary Pathobiology, Faculty of Life Sciences, University of Copenhagen, Frederiksberg, Denmark; 3 Department of Clinical Microbiology, Hvidovre Hospital, Hvidovre, Denmark; 4 Department of Clinical Microbiology, Hillerød Hospital, Hillerød, Denmark; Tulane University, The United States of America

## Abstract

*Klebsiella pneumoniae* is an important opportunistic pathogen and a frequent cause of nosocomial infections. We have characterized a *K. pneumoniae* strain responsible for a series of critical infections in an intensive care unit over a two-year period. The strain was found to be remarkably thermotolerant providing a conceivable explanation of its persistence in the hospital environment. This marked phenotype is mediated by a novel type of Clp ATPase, designated ClpK. The *clpK* gene is encoded by a conjugative plasmid and we find that the *clpK* gene alone renders an otherwise sensitive *E. coli* strain resistant to lethal heat shock. Furthermore, one third of a collection of nosocomial *K. pneumoniae* isolates carry *clpK* and exhibit a heat resistant phenotype. The discovery of ClpK as a plasmid encoded factor and its profound impact on thermal stress survival sheds new light on the biological relevance of Clp ATPases in acquired environmental fitness and highlights the challenges of mobile genetic elements in fighting nosocomial infections.

## Introduction

The Gram-negative enterobacterium *Klebsiella pneumoniae* is considered an important opportunistic pathogen frequently implicated in nosocomial infections such as urinary tract infection, pneumonia and sepsis [1]. As the cause of Gram-negative nosocomial sepsis *K. pneumoniae* is second to only *Escherichia coli* [2], however, several recent pro- and retrospective studies have even placed *Klebsiella* as the predominant causal agent of Gram-negative bacteremic cases, especially within the intensive care units (ICUs) [3]–[5]. The often immunocompromised patients suffering from severe underlying diseases in ICUs are frequently susceptible to infection with this opportunistic pathogen [6]. *K. pneumoniae* infections are associated with significant mortality depending on the site of infection i.e. systemic infection fatality rates between 20 and 50% are often reported, the rate even reaching 70% [4], [7]–[9].

*K. pneumoniae* is ubiquitous in nature and a commensal of the human gastrointestinal tract. Hence, the gastrointestinal tract is considered the main reservoir from where the bacterium is spread in the nosocomial environment, and contaminated hospital equipment and person-person contact are primary routes of transmission [10]. Virulence factors associated with *K. pneumoniae* pathogenicity include capsular polysaccharide (CPS), lipopolysaccharide (LPS) and fimbrial adhesins of type 1 and type 3, although a comprehensive understanding of *K. pneumoniae* infection mechanisms remains elusive. *K. pneumoniae* strains of environmental origin are capable of expressing the same arsenal of virulence factors as clinical isolates [11] and have comparable virulence potential when tested in animal infection models [12]. Thus, the ubiquity of *K. pneumoniae* poses a constant threat to the immunocompromised host.

Bacterial resistance to adverse conditions depends on protein members of the heat shock response [13] comprising two major classes of proteins, chaperones and proteases, both being involved in protein homeostasis and regulatory functions [14], [15]. The conserved Clp/Hsp100 ATPase protein family is traditionally divided into subfamilies based on the number of ATPase motifs as well as other structural motifs [16]–[18]. All Clp ATPases are expected to have intrinsic chaperone activity [16], [19]–[21], whereas association with ClpP confer proteolytic activity and depends on specific signature motifs [22]. The expression of different Clp proteins and the phenotype associated with their presence is species-dependent, however, a protein-threading mechanism seems to be a shared feature [16].

In this study we have identified a recurrent clinical *K. pneumoniae* strain with a remarkably increased heat resistance. We show that the thermotolerance phenotype is attributable to a novel plasmid encoded Clp ATPase, designated ClpK, and that the presence of this *clpK* gene correlates positively with the thermotolerance phenotypes observed among clinical isolates.

## Results and Discussion

### The nosocomial strain *K. pneumoniae C132-98* exhibits a heat resistant phenotype

**Figure 1:**
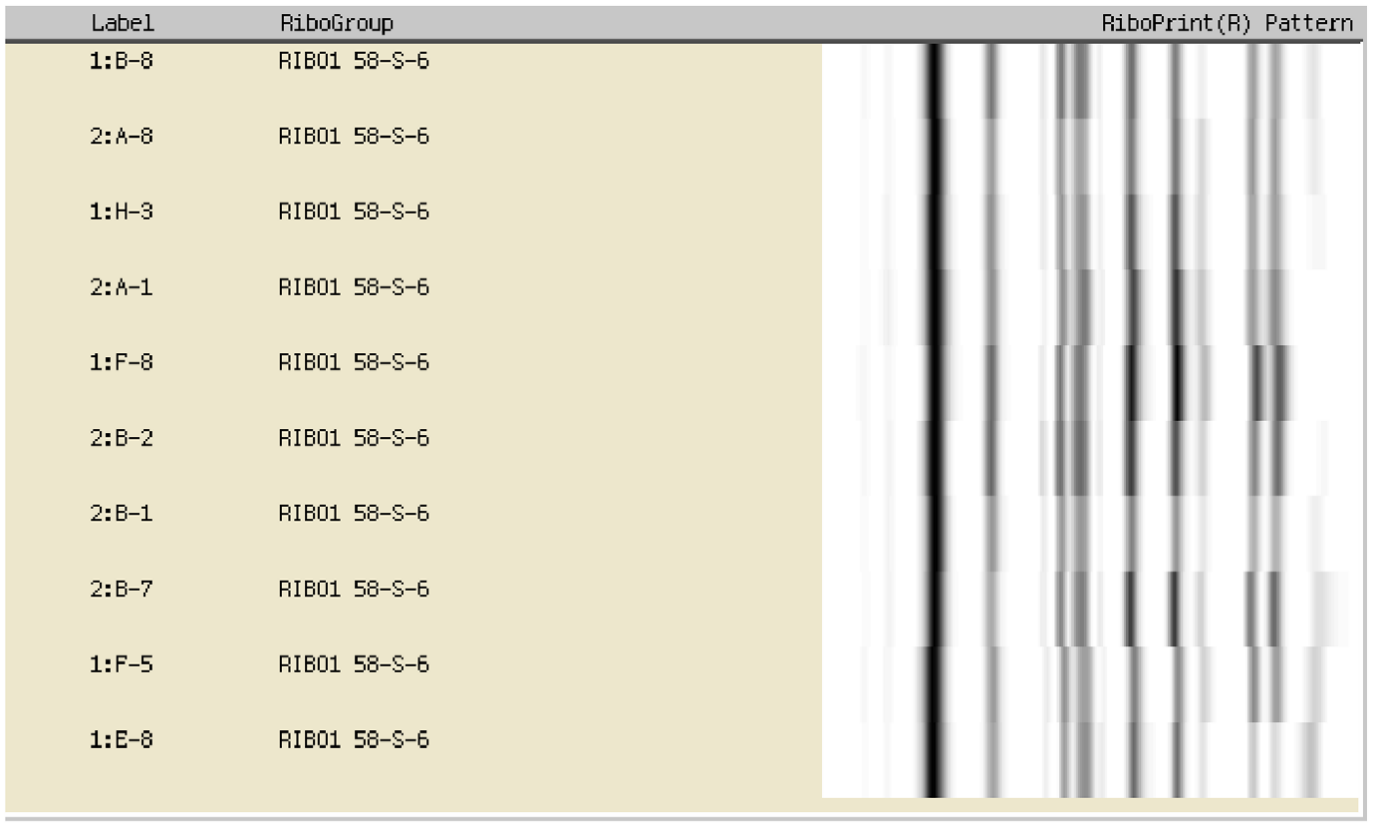
Verification of strain clonality among clinical *Klebsiella* pneumoniae isolates. 10 independent ICU isolates collected over a two-year period are positioned within the same RiboGroup as shown by identical EcoRI RiboPrint patterns.

During the course of a surveillance study at a Danish hospital, we noticed a series of ICU infections caused by capsular serotype K28 *K. pneumoniae*. All isolates were resistant to gentamicin, netilmicin, tobramycin and sulphonamide. Additionally, two isolates exhibited an ESBL (extended-spectrum beta-lactamase) phenotype as revealed by clavulanic acid reversible ceftazidime resistance. These strains were isolated from at least 10 severe cases (nine cases being fatal) over a two-year period. The clonal relatedness of the isolates was confirmed by ribotyping using the restriction enzyme EcoRI (Fig. 1), which has a high discriminatory power for *K. pneumoniae* [23], [24]. The recurring clone of *K. pneumoniae* is represented by strain C132-98. Source and route of transmission was never proven but, interestingly, infections caused by this clone ceased concomitantly with an increased focus on the cleaning/disinfection of reusable flexible endoscopes. We have previously shown that *K. pneumoniae* C132-98 is not more virulent compared to other clinical strains and isolates of environmental origin [12]. Thus, the repeated emergence of C132-98 was not directly attributable to a highly virulent phenotype.

**Figure 2:**
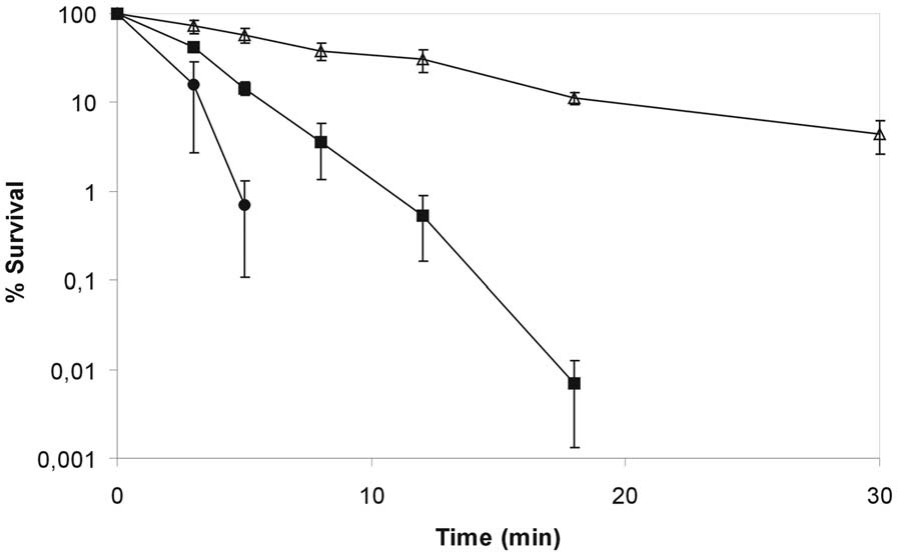
*K. pneumoniae* C132-98 exhibits a remarkably high heat shock tolerance. The tolerance to heat shock at 55°C of C132-98 (Δ) compared to the clinical strain *K. pneumoniae* C3091 (▪) and the reference strain for capsule serotype K28 (•). Means and standard error of the means from four independent experiments are shown.

Therefore, we initiated a search for increased resistance to environmental stressors that could explain the persistence of this specific strain in the nosocomial environment and found that C132-98 exhibits a markedly higher heat tolerance when compared to another clinical strain *K. pneumoniae* C3091 and the reference strain for *K. pneumoniae* capsule serotype K28. When shifted from 37°C to 55°C, strain C132-98 was detectable by plating for more than 30 minutes after the shift, whereas the other strains were undetectable already 15 minutes after the shift (Fig. 2). Even when exposed to 58°C or 60°C, C132-98 showed prolonged survival compared to the control strains (not shown).

Thus, the remarkable heat resistance of *K. pneumoniae* C132-98 may provide a plausible explanation for the repeated emergence of this specific strain within the given ICU, especially when taken into consideration that the standard disinfection procedure of flexible endoscopes is a combined thermo-chemical treatment below 60°C due to the thermo-lability of the endoscopes, combined with the extensive use of such endoscopes within ICUs. Our hypothesis is further supported by the virulence study [12] that excluded the possibility of explaining the clinical history of C132-98 by a specifically virulent phenotype.

### Isolation and identification of *clpK*

**Figure 3:**
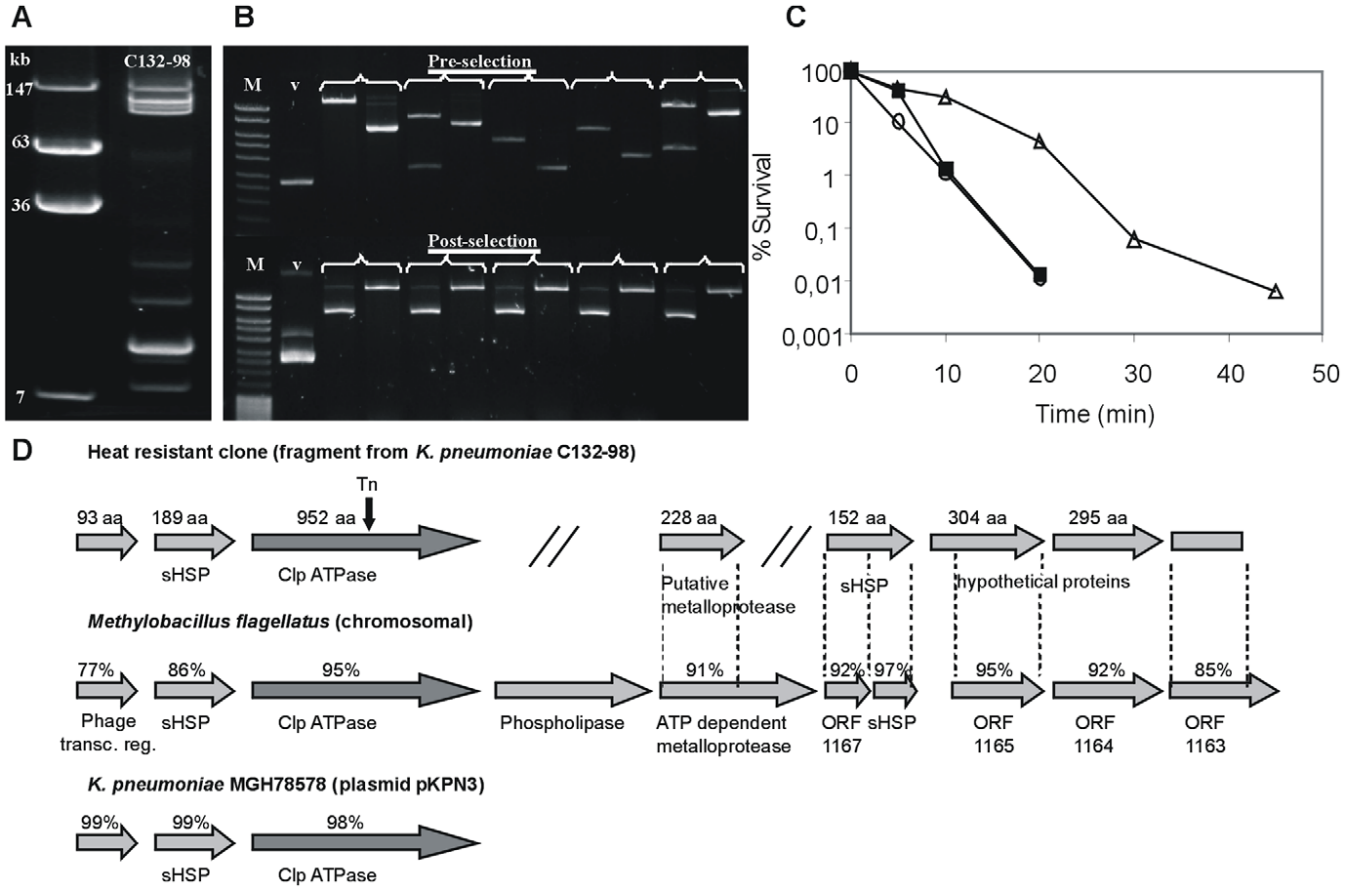
Isolation and identification of a Clp ATPase involved in the heat resistance of *K. pneumoniae* C132-98. (A) Plasmid profile of C132-98 compared to reference molecular marker of known molecular mass. (B) C132-98 plasmid DNA library construction and thermal selection. Verification of clonal diversity of library constructs by EcoRI restriction analysis of 5 representative clones (cut and uncut plasmids, respectively) prior to thermal selection. Selection of a specific clone (pMB58) as shown by similar EcoRI restriction patterns of 5 representative colonies (uncut and cut plasmids, respectively) obtained from the pooled library following repeated heat shock treatments. (M) Molecular marker <10 kb, (v) empty vector. (C) Assessment of thermal protection of *E. coli* DH_5_α at 53°C conferred by plasmid pMB58 (Δ) compared to the empty vector background pBR322 (○). A derivative of pMB58 subjected to in vitro transposon mutagenesis, pMB58_Tn (▪), shows basal thermal protection comparable to the empty vector. One representative experiment out of several is shown. (D) Seven putative ORFs identified on the cloned fragment (pMB58) from C132-98 plasmid DNA. Identities of deduced amino acids to a chromosomal fragment of *M. flagellatus* and a locus on plasmid pKPN3 from *K. pneumoniae* MGH78578 are given. The position of the transposon insertion in pMB58_Tn within the Clp ATPase is indicated.

A search for the genetic locus involved in the heat resistance of *K. pneumoniae* C132-98 was conducted. Plasmid profiling revealed that C132-98 possesses several plasmids (≥eight). Four of them are large plasmids equal to or larger than 100 kb (Fig. 3A). Thus, the thermo-protective genetic locus could be located on a plasmid. To examine this notion, we constructed a DNA library from C132-98 plasmid DNA (Fig. 3B) and, anticipating that the heat resistant property would be expressed in *E. coli*, a total of 384 library clones were pooled and subjected to thermal selection. Hereby, a specific plasmid, pMB58, was selected conferring increased heat resistance (Fig. 3B) also after reintroduction into a clean *E. coli* DH_5_α background (Fig. 3C). The plasmid carrying strain was detectable up to 45 min at 53°C compared to the vector control strain being undetectable after 20 min.

DNA sequencing of pMB58 revealed an 8187 bp fragment (GenBank accession FJ042668) covering seven putative open reading frames (ORFs). The segment shows a high overall similarity to a chromosomal region from the *Methylobacillus flagellatus* genome (GenBank accession NC_007947). Three of the ORFs are also homologous to a segment of a plasmid encoded sequence (pKPN3) found in *K. pneumoniae* MGH78578 (GenBank accession NC_009649) (Fig. 3D). The cloned segment covers genes corresponding to, among others, putative small heat shock proteins (sHSP) and a Clp ATPase. In order to identify the gene responsible for the heat resistant phenotype, in vitro transposon mutagenesis of pMB58 was performed and a mutant exhibiting a thermal tolerance comparable to *E. coli* carrying the empty vector was identified (Fig. 3C). The mutagenesis revealed that the ORF corresponding to the putative Clp ATPase gene was responsible for the heat resistance mediated by pMB58 in *E. coli* DH_5_α. The protein corresponding to the Clp ATPase gene was designated ClpK (K for *Klebsiella*).

**Figure 4:**
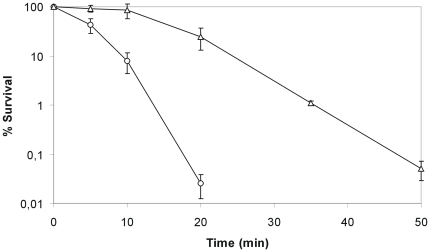
The identified ClpK ATPase is involved in tolerance to thermal stress. Heat shock resistance in *E. coli* DH_5_α at 53°C conferred by pClpK (Δ) encoding the ClpK ATPase gene from *K. pneumoniae* C132-98 compared to the empty vector background pACYC184 (○).Means and standard error of the means from three independent experiments are shown.

The implication of the *clpK* gene in thermal protection was further confirmed by expressing ClpK (pClpK) in *E. coli* leading to survival of approximately 10% of the cells after 20 min of heat shock at 53°C compared to a 100-fold reduction (0.1%) in the absence of ClpK (Fig. 4).

### ClpK is necessary for the heat tolerance of *K. pneumoniae C132-98*

**Figure 5:**
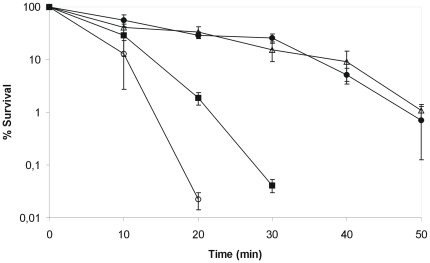
The *clpK* gene is necessary for thermal tolerance of *K. pneumoniae* C132-98. Survival rates of wild type C132-98 (Δ) during heat shock at 53°C compared to an isogenic Δ*clpK* mutant (○), and the Δ*clpK* mutant complemented with pClpK (▪) or pMB58-sub (•), respectively. Means and standard error of the means from four independent experiments are shown.

A Δ*clpK* mutation was constructed in *K. pneumoniae* C132-98 by allelic replacement. When exposed to heat, viable *K. pneumoniae* were detectable for at least 50 min at 53°C, whereas the CFU of the Δ*clpK* mutant was below the detection limit after 20 min exposure (Fig. 5). The reduced heat tolerance of the C132-98 Δ*clpK* mutant strain could be complemented in part by plasmid pClpK expressing ClpK (Fig. 5) and therefore, we conclude that ClpK is involved in the heat resistance of *K. pneumoniae* C132-98. Since the Δ*clpK* mutant was incompletely complemented by pClpK, a polar effect due to the deletion was considered. Indeed, transformation of the entire heat resistance locus (cf. Fig. 3D) by plasmid pMB58-sub fully restored the thermal tolerance of C132 Δ*clpK* (Fig. 5) Therefore, obstruction of *clpK* in C132 most likely lead to malfunction of co-localized genes involved in heat resistance. Interestingly, genes encoding small heat shock proteins (sHSP) are present up- and down-stream *clpK*. In *E. coli* the sHSPs IbpA/IbpB (inclusion body-associated proteins) bind intracellular proteins aggregated by heat shock and allow refolding in a process involving the ClpB ATPase [25]–[29]. A similar collaboration between ClpK and the products from these co-localized sHSP genes in stress alleviation is possible. At least, given their co-localization, it is tempting to speculate that these genes may be functionally related. However, the thermoprotective activity of ClpK is most likely not strictly dependent on any co-localized genes since *clpK* alone exerts a heat resistance phenotype when transformed into *E. coli* and significantly enhances the heat resistance of C132 Δ*clpK*. Further studies are needed to identify the specific genes involved in ClpK-mediated heat resistance and to assess any functional collaboration.

No other specific phenotypes, e.g., sensitivity to high salt (NaCl) concentrations, impact of H_2_O_2_ on growth, and survival under desiccative conditions were observed for the Δ*clpK* mutant strain when compared to the wild type. Moreover, the maximum temperature allowing growth (46°C) was unaffected by the Δ*clpK* mutation (not shown). Thus, the *clpK* locus seems to specifically enhance the ability of C132-98 to survive an otherwise lethal temperature.

### *clpK* is encoded on a conjugative plasmid

**Figure 6:**
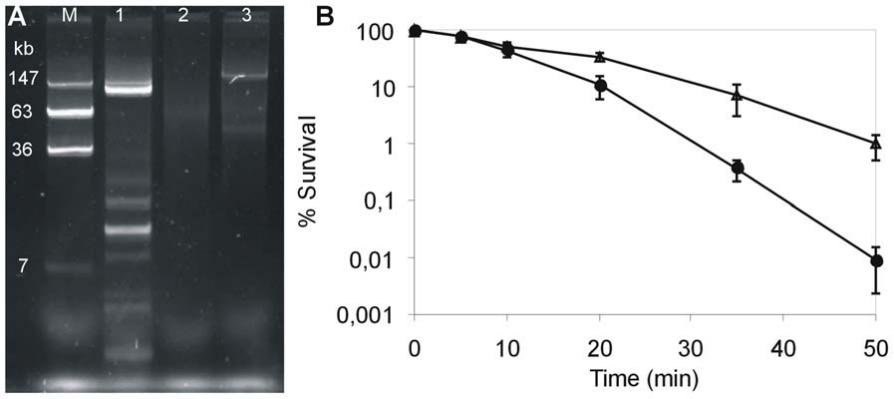
A plasmid encoded thermotolerance locus from *K. pneumoniae* C132-98 is transferable by conjugation rendering *E. coli* MG1655 heat resistant. (A) Plasmid profile. Lanes 1-3 represent *K. pneumoniae* C132-98, recipient *E. coli* MG1655, and a gentamicin resistant transconjugant of *E. coli* MG1655, respectively, and lane M represents *E. coli* 39R861 harboring four plasmids as a molecular weight marker. (B) Heat shock survival at 53°C of *E. coli* MG1655 transconjugant (Δ) compared to the parental MG1655 recipient strain (•). Means and standard error of the means from three independent experiments are shown.

To confirm that the *clpK* gene is present on a plasmid we purified plasmid DNA from C132-98 Δ*clpK*, in which the *clpK* gene is substituted with a tetracycline encoding cassette; transformed *E. coli* and selected a tetracycline resistant transformant that harbored only the largest 150 kb plasmid (not shown). The presence of the *clpK* deletion on the plasmid was confirmed by PCR (not shown), demonstrating that *clpK* is indeed encoded by the largest plasmid in *K. pneumoniae* C132-98. Consequently, we wished to establish whether *clpK* and the heat resistance phenotype was transferable by conjugation. Indeed, by simple plate mating and selection for gentamicin resistance we isolated a transconjugant of *Escherichia coli* MG1655 (Fig. 6A) with a notably heat resistant phenotype. In fact, the recipient strain exhibited an approximately 100-fold improved survival after 50 minutes at 53°C (Fig. 6B). A few plasmid encoded Clp ATPases have previously been identified; however, no significant phenotypes were associated with their presence or inactivation [30], [31]. We believe that the present study provides the first reported example of a plasmid encoded Clp ATPase having a clear phenotypic effect demonstrated by an improved tolerance to thermal stress.

### *ClpK* defines a novel subclass of *Clp ATPases*

**Figure 7:**
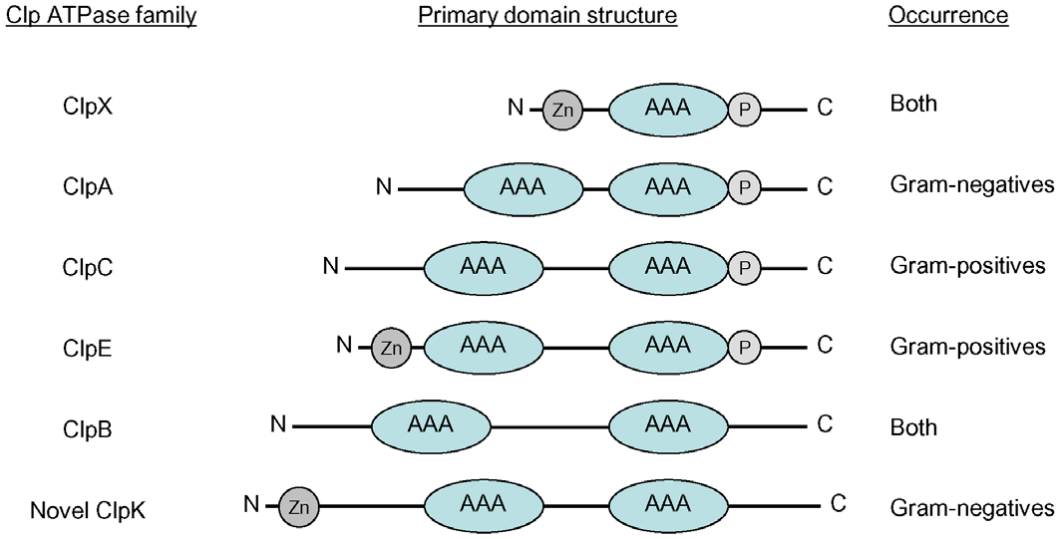
Major defining features separating the novel ClpK protein from established Clp ATPase families. ClpK is characterized by a unique extended N-terminal, the presence of a putative zinc finger motif (Zn), having a linker domain of intermediate length, and lacks a ClpP interaction tripeptide (P).

Alignments of ClpK with established members of the Clp ATPase family revealed that ClpK displays primary amino acid similarity to the class I Clp ATPases carrying two nucleotide binding domains (cf. Fig. 7). Uniquely, ClpK carries a 100 amino acid N-terminal extension not observed in other Clp proteins, and the linker domain separating the nucleotide-binding domains is of intermediate size resembling ClpC and its relatives, ClpE and ClpL, that are considered Gram-positive Clp ATPases only. ClpK is clearly distinct from the previously recognized Gram-negative Clp ATPases, ClpA and ClpB, carrying small and large linker domains, respectively. Thus, this is the first report on a Gram-negative Clp ATPase having a linker domain of intermediate size.

The amino acid sequence of ClpK shares extensive identity with uncharacterized Clp proteins in completed genomes from *E. coli* (98%), *Enterobacter cloacae* (98%), *M. flagellatus* (95%), *Desulfovibrio desulfuricans* (94%), *Comamonas testosteroni* (93%), *Marinobacter aquaeolei* (92%), *Pseudomonas mendocina* (90%) and *Ochrobactrum anthropi* (73%) among others. Most intriguingly, these bacteria represent species from all proteobacterial subdivisions (α, β, Δ and γ) except division ε. Thus, ClpK is more likely to have evolved by horizontal transfer rather than vertical inheritance. Of additional interest, ClpK homologous proteins are identified in species such as *Silicibacter lacuscaerulensis* and *Dictyoglomus thermophilum* considered moderately thermotolerant mesophile and bona fide thermophile species, respectively. We did not identify ClpK homologues in any Gram-positive species, suggesting that this ATPase is unique to Gram-negative bacteria.

By comparing the available amino acid sequences of ClpK homologues, other distinct features were identified. These include the absence of a characteristic tripeptide that is proposed to be required for the Clp ATPase partners to interact with the proteolytic subunit ClpP in *E. coli* and other bacteria [32] and the presence of three conserved cysteine and a single histidine residue in the amino terminal domain possibly forming a zinc finger motif or a reminiscent thereof. Zinc finger motifs are found in ClpX and in ClpE where it is functionally important [33], [34]. The significance of the identified motif in ClpK remains to be elucidated, however, the high interspecies amino acid similarity within the N-terminal 250 amino acids of ClpK proteins (>85%) suggests that this domain exerts functional properties essential and/or unique to ClpK.

Defining features of ClpK compared to established subclasses of Clp proteins are depicted in Figure 7. We named the protein ClpK as it constitutes a separate subclass within the Clp ATPase family and *K. pneumoniae* ClpK is the prototype of this novel subclass.

### Prevalence of clpK in clinical *K. pneumoniae strains* and correlation with heat resistant phenotype

**Figure 8:**
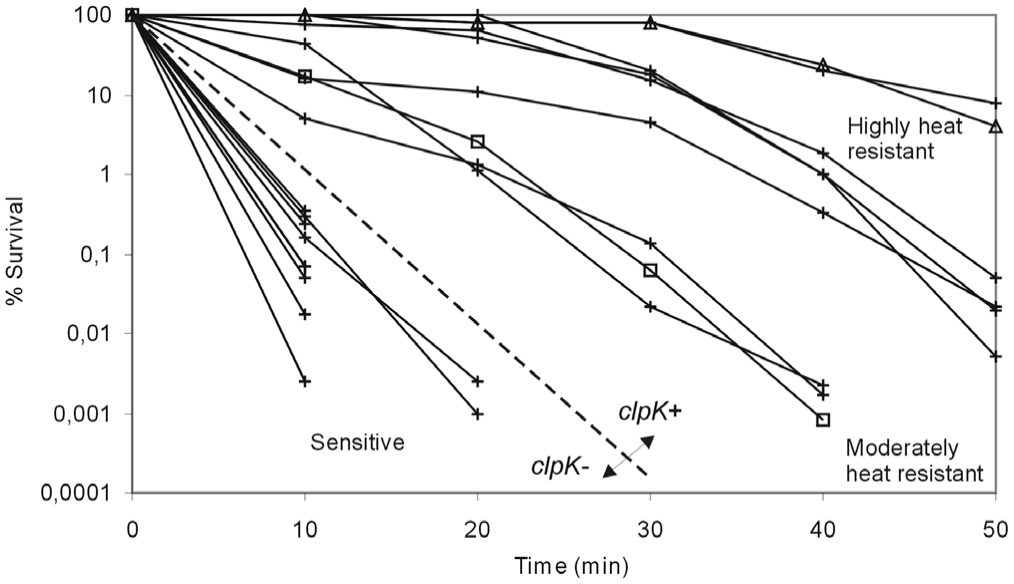
The presence of *clpK* correlates with a heat shock resistant phenotype of clinical *K. pneumoniae*. Survival potential during heat shock at 53°C evaluated on nine clinical *K. pneumoniae* strains, including C132-98 (Δ) and MGH78578 (□), being positive for *clpK* (determined by colony blot hybridization) compared to nine *clpK* negative strains. The two groups are separated by the discontinuous line.

In order to determine the prevalence of *clpK* among *K. pneumoniae* strains, a collection of 105 clinical isolates of *K. pneumoniae* was tested using a nucleotide probe targeting the conserved linker domain sequence unique to *clpK*. By this screening, we detected *clpK* in 31 (30%) of the strains (not shown). Hence, *clpK* is not unique to strain C132-98, but the gene is present in a subset of clinical *K. pneumoniae* isolates. Consequently, the thermotolerance of a randomly chosen representative collection of *clpK* positive and negative *K. pneumoniae* strains was compared (Fig. 8) and a positive correlation between having the *clpK* gene and expressing a thermotolerant phenotype was established. Of the strains tested, all strains devoid of *clpK* were undetectable after 20 min of heat shock at 53°C, whereas all *clpK* positive strains exhibited detectable survival at 40 min and beyond. A marked strain-to-strain difference in heat resistance was observed, which may be due to the presence/absence of other genes coexisting with *clpK*, such as the downstream sHSP found in C132-98 which are absent in MGH78578 (cf. Fig. 3D). Indeed, our complementation experiment (Fig. 5) showed that other factors encoded at the *clpK* locus are of importance in resistance toward thermal stress. Thus, high level heat resistance requiring more than the ClpK protein alone seems a plausible reason for observed differences in resistance phenotypes among isolates. Alternatively, ClpK expression may also differ between strains. Nevertheless, a clear association between the presence of the *clpK* gene and elevated thermal tolerance was established for *K. pneumoniae*.

### Concluding remarks

A *K. pneumoniae* strain being considerably resistant towards thermal stress was identified in a hospital setting. Molecular cloning and phenotypic studies allowed identification of a novel plasmid encoded ClpK ATPase mediating the increased heat resistance. In previous studies, chromosomally encoded Clp ATPases have been demonstrated to be required for growth or survival at high temperature such as the ClpB protein in *E. coli* [35] and *Staphylococcus aureus* [36]; and the ClpC in *Listeria monocytogenes* [37] and *S. aureus* [36] among others. This study is the first to report on a novel Clp ATPase that, as an accessory factor expressed from a plasmid, enhances the heat tolerance of the host strain and greatly increases survival under conditions that are found in the hospital setting. The *clpK* gene as a predictor of elevated thermal tolerance in *K. pneumoniae* is an interesting concept that may be determining in the ability of this opportunistic pathogen to survive and persist in niches of the nosocomial environment e.g. in reusable flexible endocopes undergoing thermo-chemical processing in which temperature is a critical parameter [38]. It can be speculated that the nosocomial persistence of *K. pneumoniae* C132-98 (and maybe other clinical isolates) was facilitated by acquisition of *clpK*.

ClpK homologues were found in genomes from *E. cloacae* and *E. coli* among others. To date, *clpK* was observed only in one genome from all the *E. coli* genomes available at NCBI, Escherichia coli MS 115-1, and in genome Escherichia sp. 1_1_43. Thus, as for *K. pneumoniae*, *clpK* is not ubiquitous in *E. coli* but present only in a subset of isolates. Since we observed that transformation of laboratory *E. coli* strains with *clpK* confer thermal resistance, it is a reasonable anticipation that ClpK exerts a heat resistant phenotype in this species, and the *clpK* gene may very well be of importance in acquired environmental fitness across several clinically relevant species.

We observed that *clpK* was transferable by conjugation which also endowed gentamicin resistance. Intriguingly, the endemic cluster from which C132-98 was isolated included clonal isolates that had acquired ESBL producing capabilities. Multiple drug resistant *K. pneumoniae* producing ESBLs are of emerging clinical significance [39] and of major concern due to poorer prognosis [40], [41]. Studies have identified mobile environmental loci as source and route of infection [42], [43] and studies have established a link between inadequately decontaminated endoscopes and a series of infections due to ESBL-producing [44] and recently carbapenem-resistant [45] *K. pneumoniae*. In this respect, the combination of multiple antibiotic resistance and bacterial factors increasing environmental fitness may prove to be of significant importance in the prevention of infections and spreading of clinically relevant pathogens. Bacterial factors of clinical relevance, such as virulence genes and antibiotic resistance genes, are often situated on plasmids. Thus, a compelling scenario is the probable co-transfer of such genes with genes enhancing environmental fitness which would be highly advantageous to clinical strains residing in the hospital setting.

## Materials and Methods

### Strains and growth conditions

The clinical *K. pneumoniae* strains used in this study were all obtained from The International *Escherichia* and *Klebsiella* Reference Centre (WHO), Statens Serum Institut. 105 bacteremic isolates previously serotyped according to CPS antigens by counter-current immuno-electrophoresis [9] and the ICU blood isolate *K. pneumoniae* C132-98 [12] were collected from Hvidovre Hospital, Denmark. The clinical strain *K. pneumoniae* C3091 [46], *K. pneumoniae* strain MGH78578 (ATCC700721) and the serotype K28 type strain 5758 [47] were included as references. *E. coli* 39R861 [48] was used as a standard reference for plasmid size estimation. *E. coli* DH_5_α was used for cloning purposes. All strains were grown in Luria-Bertani (LB) medium at 37°C. Transformation of *E. coli* and *K. pneumoniae* was performed by electroporation followed by selection on LB plates supplemented with appropriate antibiotics at the following concentrations: ampicillin (100 µg ml^−1^), apramycin (30 µg ml^−1^), chloramphenicol (12.5 µg ml^−1^), gentamicin (20 µg ml^−1^), and tetracycline (8 µg ml^−1^).

### DNA manipulations and plasmid constructions

Molecular typing of isolates was carried out by using a RiboPrinter™ and restriction enzyme EcoRI. Extraction of *K. pneumoniae* C132-98 endogenous plasmids was performed by a procedure in essence as previously described [49]. Purification of large endogenous plasmids for cloning purposes was performed with Plasmid Mini AX DNA (DNA-Gdañsk). Cloned plasmid DNA was purified using the Qiaprep Spin Miniprep Kit (Qiagen). DNA restriction enzyme digestions and phosphatase treatment (Antarctic Phosphatase) were performed according to the manufacturer's instructions (New England Biolabs). Ligation was performed by the Fast-Link DNA Ligation Kit (EPICENTRE Biotechnologies). In vitro transposon mutagenesis was carried out by the EZ-Tn5™ <*oriV*/KAN-2> Insertion Kit as recommended (EPICENTRE Biotechnologies). Polymerase chain reactions (PCR) were conducted with Expand High Fidelity PCR Kit and PCR DIG Probe Synthesis Kit (both Roche) under standard conditions according to the manual. Primers were obtained from and sequencing carried out at MWG Biotech AG.

Plasmid pBR322 was used for the construction of plasmid DNA library in *E. coli* DH_5_α. Purified plasmid DNA from *K. pneumoniae* C132-98 was partially digested with Sau3AI and size-fractionated by agarose electrophoresis from which 4–10 kbp fragments were purified. Following ligation with a BamHI linearized dephosphorylated pBR322 vector and transformation of electro-competent *E. coli*, a total of 384 library clones were picked and held in microtiter trays. Plasmid pACYC184 was used for subcloning and expression in *E. coli* and *K. pneumoniae*. The entire heat resistance locus from pMB58 (pBR322) was cloned into pACYC184, generating pMB58-sub, using enzymes NheI and SalI to allow transformation of *K. pneumoniae* (intrinsically ampicillin resistant). The *clpK* encoding region was cloned by PCR using primer pair F_clpk (5′-GGGGGGGGATCCGAACCTGCCTGACGACGCCAACCA-3′)/R_clpk (5′-GGGGGGGTCGACGCAGCCTCGCCCGCCATCAAGA-3′) equipped with BamHI and SalI restriction recognition sites, respectively, generating pClpK.

### Construction of an isogenic clpK deletion mutant

The *clpK* gene in *K. pneumoniae* C132-98 was deleted by allelic exchange with a PCR synthesized cassette encoding tetracycline resistance flanked by regions homologous to sequences up- and down-stream the plasmid encoded *clpK* gene by a modification of the lambda Red mediated recombination procedure [50] previously applied to chromosomal gene replacement in *K. pneumoniae* [51]. Allelic replacement was aided by the thermo-sensitive helper plasmid pKOBEGApra, an apramycin resistant derivative of pKOBEG [52], encoding lambda Red recombinase functions. Plasmid pKOBEGApra bearing C132-98 was grown at 30°C, induced by addition of 0.2% arabinose (Sigma), and cured of the plasmid at 37°C. Initially, the tetracycline resistance encoding cassette was amplified from pAR82 using the primer pair Ucas (5′-CAAGAATTGCCGGCGGAT-3′)/Dcas (5′-GGTATTTCACACCGCATAGC-3′) [53]. In addition, 528 bp and 569 bp regions flanking the *clpK* gene was amplified from C132-98 plasmid DNA by use of primer pair F_upclpK (5′-GCCGGTGCAGCGCAATGACCT-3′)/R_upclpK (5′-ATCCGCCGGCAATTCTTGCGTGCCGGGTTTTTCTTGTGACGA-3′) and F_dwnclpK (5′-GCTATGCGGTGTGAAATACCTCGGCGAGCGACGCATCTT-3′)/R_dwnclpK (5′-CTCCAACACGCGGGCATAGG-3′), respectively. At their 5′ ends, primers R_upclpK and F_dwnclpK were equipped with 18 bp and 20 bp regions homologous to the extremities of the tetracycline resistance cassette. Subsequently, the deletion fragment was generated by PCR using the primer combination F_upclpK/R_dwnclpK and equimolar amounts of all three fragments as template. The purified PCR product was introduced to electro-competent C132-98 harboring pKOBEGApra followed by tetracycline selection and temperature-induced curing of pKOBEGApra. Correct allelic replacement was verified by PCR.

### Heat shock assays

All heat shock assays were conducted by dilution of stationary phase bacterial cultures 1/10 in pre-heated 0.9% saline (NaCl) solutions at indicated temperatures until sample withdrawal at relevant time intervals. Selection of the Sau3AI generated plasmid DNA library was performed by 5 min of heat shock of the pooled library clones (384 individual clones) at 58°C followed by reinoculation into fresh LB media. Following outgrowth of the pooled bacteria the combined heat shock and outgrowth procedure was repeated three times. Clonal selection was verified by plasmid restriction analysis of representative randomly picked colonies after growth on LB plates. Heat shock survival curves of individual strains or clones at indicated temperatures were generated by sample withdrawal at appropriate time intervals and the survival rates were estimated by determination of CFU relative to the bacterial counts prior to heat shock.

### Hybridization experiments

105 bacteremic isolates of *K. pneumoniae* were screened for presence of the *clpK* gene by colony blot hybridization on Amersham Hybond™-N+ membranes (GE Healthcare). A 437 bp digoxigenin (DIG)-labeled probe was PCR synthesized from *K. pneumoniae* C132-98 plasmid DNA using primers F_clpKprobe (5′-CGGCCTGCGCGACACCTT-3′) and R_clpKprobe (5′-TTTCGCGCTCTTCCACCGTCAACT-3′) directed against the sequence corresponding to the unique and conserved linker domain of *clpK*. Stringent hybridization conditions were applied and immunological detection of *clpK* positive isolates was performed by the DIG Nucleic Acid Detection Kit according to the directions of the manufacturer (Roche). *K. pneumoniae* C132-98 and *K. pneumoniae* C132-98 Δ*clpK* were used as positive and negative controls, respectively.

### Database comparisons and sequence analysis

The nucleotide sequence data reported in this work have been deposited in the GenBank database under accession number FJ042668. Nucleotide sequence analysis was performed by using the Lasergene v. 5.07 software (DNAStar, Inc.). NCBI BLAST searches were performed applying default parameter values [54]. Multiple sequence alignments were conducted by the ClustalW2 algorithm provided by EMBL-EBI [55].

### Accession numbers of sequences and proteins mentioned in the text

*Escherichia coli* ClpK, EFJ96502; *Escherichia sp.* ClpK, EEH72429; *Enterobacter cloacae* ClpK, ADF63230; *Klebsiella pneumoniae* C132-98 heat resistance locus, FJ042668; *K. pneumoniae* MGH78578 plasmid pKPN3, NC_009649; *Methylobacillus flagellatus* genome, NC_007947; *K. pneumoniae* MGH78578 ClpK, ABR80278; *M. flagellatus* ClpK, ABE49438; *Desulfovibrio desulfuricans* ClpK, ABB40206; *Comamonas testosteroni* ClpK, EED65962; *Marinobacter aquaeolei* ClpK, ABM20464; *Pseudomonas mendocina* ClpK, ABP85128; *Ochrobactrum anthropi* ClpK, ABS17166.

## References

[1] Podschun R, Ullmann U (1998). *Klebsiella* spp. as nosocomial pathogens: epidemiology, taxonomy, typing methods, and pathogenicity factors.. Clin Microbiol Rev.

[2] Sahly H, Podschun R (1997). Clinical, bacteriological, and serological aspects of *Klebsiella* infections and their spondylarthropathic sequelae.. Clin Diagn Lab Immunol.

[3] Couto RC, Carvalho EA, Pedrosa TM, Pedroso ER, Neto MC (2007). A 10-year prospective surveillance of nosocomial infections in neonatal intensive care units.. Am J Infect Control.

[4] Ghotaslou R, Ghorashi Z, Nahaei MR (2007). *Klebsiella pneumoniae* in neonatal sepsis: a 3-year-study in the pediatric hospital of Tabriz, Iran.. Jpn J Infect Dis.

[5] Pérez-González LF, Ruiz-González JM, Noyola DE (2007). Nosocomial bacteremia in children: a 15-year experience at a general hospital in Mexico.. Infect Control Hosp Epidemiol.

[6] Sahly H, Podschun R, Ullmann U (2000). *Klebsiella* infections in the immunocompromised host.. Adv Exp Med Biol.

[7] García de la Torre M, Romero-Vivas J, Martínez-Beltrán J, Guerrero A, Meseguer M (1985). *Klebsiella* bacteremia: an analysis of 100 episodes.. Rev Infect Dis.

[8] Feldman C, Smith C, Levy H, Ginsburg P, Miller SD (1990). *Klebsiella pneumoniae* bacteraemia at an urban general hospital.. J Infect.

[9] Hansen DS, Gottschau A, Kolmos HJ (1998). Epidemiology of *Klebsiella* bacteraemia: a case control study using *Escherichia coli* bacteraemia as control.. J Hosp Infect.

[10] Montgomerie JZ (1979). Epidemiology of *Klebsiella* and hospital-associated infections.. Rev Infect Dis.

[11] Podschun R, Pietsch S, Höller C, Ullmann U (2001). Incidence of *Klebsiella* species in surface waters and their expression of virulence factors.. Appl Environ Microbiol.

[12] Struve C, Krogfelt KA (2004). Pathogenic potential of environmental *Klebsiella pneumoniae* isolates.. Environ Microbiol.

[13] Guisbert E, Yura T, Rhodius VA, Gross CA (2008). Convergence of molecular, modeling, and systems approaches for an understanding of the *Escherichia coli* heat shock response.. Microbiol Mol Biol Rev.

[14] Wickner S, Maurizi MR, Gottesman S (1999). Posttranslational quality control: folding, refolding, and degrading proteins.. Science.

[15] Jenal U, Hengge-Aronis R (2003). Regulation by proteolysis in bacterial cells.. Curr Opin Microbiol.

[16] Zolkiewski M (2006). A camel passes through the eye of a needle: protein unfolding activity of Clp ATPases.. Mol Microbiol.

[17] Squires C, Squires CL (1992). The Clp proteins: proteolysis regulators or molecular chaperones?. J Bacteriol.

[18] Schirmer EC, Glover JR, Singer MA, Lindquist S (1996). HSP100/Clp proteins: a common mechanism explains diverse functions.. Trends Biochem Sci.

[19] Wickner S, Gottesman S, Skowyra D, Hoskins J, McKenney K (1994). A molecular chaperone, ClpA, functions like DnaK and DnaJ.. Proc Natl Acad Sci U S A.

[20] Wawrzynow A, Wojtkowiak D, Marszalek J, Banecki B, Jonsen M (1995). The ClpX heat-shock protein of *Escherichia coli*, the ATP-dependent substrate specificity component of the ClpP-ClpX protease, is a novel molecular chaperone.. EMBO J.

[21] Schlothauer T, Mogk A, Dougan DA, Bukau B, Turgay K (2003). MecA, an adaptor protein necessary for ClpC chaperone activity.. Proc Natl Acad Sci U S A.

[22] Singh SK, Rozycki J, Ortega J, Ishikawa T, Lo J (2001). Functional domains of the ClpA and ClpX molecular chaperones identified by limited proteolysis and deletion analysis.. J Biol Chem.

[23] Bingen EH, Desjardins P, Arlet G, Bourgeois F, Mariani-Kurkdjian P (1993). Molecular epidemiology of plasmid spread among extended broad-spectrum beta-lactamase-producing *Klebsiella pneumoniae* isolates in a pediatric hospital.. J Clin Microbiol.

[24] Brisse S, Verhoef J (2001). Phylogenetic diversity of *Klebsiella pneumoniae* and *Klebsiella oxytoca* clinical isolates revealed by randomly amplified polymorphic DNA, *gyrA* and *parC* genes sequencing and automated ribotyping.. Int J Syst Evol Microbiol.

[25] Laskowska E, Wawrzynów A, Taylor A (1996). IbpA and IbpB, the new heat-shock proteins, bind to endogenous *Escherichia coli* proteins aggregated intracellularly by heat shock.. Biochimie.

[26] Veinger L, Diamant S, Buchner J, Goloubinoff P (1998). The small heat-shock protein IbpB from *Escherichia coli* stabilizes stress-denatured proteins for subsequent refolding by a multichaperone network.. J Biol Chem.

[27] Mogk A, Schlieker C, Friedrich KL, Schönfeld HJ, Vierling E (2003). Refolding of substrates bound to small Hsps relies on a disaggregation reaction mediated most efficiently by ClpB/DnaK.. J Biol Chem.

[28] Mogk A, Deuerling E, Vorderwülbecke S, Vierling E, Bukau B (2003). Small heat shock proteins, ClpB and the DnaK system form a functional triade in reversing protein aggregation.. Mol Microbiol.

[29] Matuszewska M, Kuczyńska-Wiśnik D, Laskowska E, Liberek K (2005). The small heat shock protein IbpA of *Escherichia coli* cooperates with IbpB in stabilization of thermally aggregated proteins in a disaggregation competent state.. J Biol Chem.

[30] Huang DC, Huang XF, Novel G, Novel M (1993). Two genes present on a transposon-like structure in *Lactococcus lactis* are involved in a Clp-family proteolytic activity.. Mol Microbiol.

[31] Suokko A, Savijoki K, Malinen E, Palva A, Varmanen P (2005). Characterization of a mobile *clpL* gene from *Lactobacillus rhamnosus*.. Appl Environ Microbiol.

[32] Kim YI, Levchenko I, Fraczkowska K, Woodruff RV, Sauer RT (2001). Molecular determinants of complex formation between Clp/Hsp100 ATPases and the ClpP peptidase.. Nat Struct Biol.

[33] Banecki B, Wawrzynow A, Puzewicz J, Georgopoulos C, Zylicz M (2001). Structure-function analysis of the zinc-binding region of the ClpX molecular chaperone.. J Biol Chem.

[34] Varmanen P, Vogensen FK, Hammer K, Palva A, Ingmer H (2003). ClpE from *Lactococcus lactis* promotes repression of CtsR-dependent gene expression.. J Bacteriol.

[35] Tomoyasu T, Mogk A, Langen H, Goloubinoff P, Bukau B (2001). Genetic dissection of the roles of chaperones and proteases in protein folding and degradation in the *Escherichia coli* cytosol.. Mol Microbiol.

[36] Frees D, Chastanet A, Qazi S, Sørensen K, Hill P (2004). Clp ATPases are required for stress tolerance, intracellular replication and biofilm formation in *Staphylococcus aureus*.. Mol Microbiol.

[37] Rouquette C, Ripio MT, Pellegrini E, Bolla JM, Tascon RI (1996). Identification of a ClpC ATPase required for stress tolerance and in vivo survival of *Listeria monocytogenes*.. Mol Microbiol.

[38] Zühlsdorf B, Winkler A, Dietze B, Floss H, Martiny H (2003). Gastroscope processing in washer-disinfectors at three different temperatures.. J Hosp Infect.

[39] Paterson DL, Ko WC, Von Gottberg A, Mohapatra S, Casellas JM (2004). International prospective study of *Klebsiella pneumoniae* bacteremia: implications of extended-spectrum beta-lactamase production in nosocomial Infections.. Ann Intern Med.

[40] Schwaber MJ, Navon-Venezia S, Kaye KS, Ben-Ami R, Schwartz D (2006). Clinical and economic impact of bacteremia with extended- spectrum-beta-lactamase-producing Enterobacteriaceae.. Antimicrob Agents Chemother.

[41] Cordery RJ, Roberts CH, Cooper SJ, Bellinghan G, Shetty N (2008). Evaluation of risk factors for the acquisition of bloodstream infections with extended-spectrum beta-lactamase-producing *Escherichia coli* and *Klebsiella* species in the intensive care unit; antibiotic management and clinical outcome.. J Hosp Infect.

[42] Gaillot O, Maruéjouls C, Abachin E, Lecuru F, Arlet G (1998). Nosocomial outbreak of *Klebsiella pneumoniae* producing SHV-5 extended-spectrum beta-lactamase, originating from a contaminated ultrasonography coupling gel.. J Clin Microbiol.

[43] van 't Veen A, van der Zee A, Nelson J, Speelberg B, Kluytmans JA (2005). Outbreak of infection with a multiresistant *Klebsiella pneumoniae* strain associated with contaminated roll boards in operating rooms.. J Clin Microbiol.

[44] Branger C, Bruneau B, Lesimple AL, Bouvet PJ, Berry P (1997). Epidemiological typing of extended-spectrum beta-lactamase-producing *Klebsiella pneumoniae* isolates responsible for five outbreaks in a university hospital.. J Hosp Infect.

[45] Naas T, Cuzon G, Babics A, Fortineau N, Boytchev I (2010). Endoscopy-associated transmission of carbapenem-resistant *Klebsiella pneumoniae* producing KPC-2 beta-lactamase.. J Antimicrob Chemother.

[46] Oelschlaeger TA, Tall BD (1997). Invasion of cultured human epithelial cells by *Klebsiella pneumoniae* isolated from the urinary tract.. Infect Immun.

[47] Ørskov I, Ørskov F (1984). in *Serotyping of* Klebsiella, ed Bergan T (New York: Academic Press Inc),.

[48] Threlfall EJ, Rowe B, Ferguson JL, Ward LR (1986). Characterization of plasmids conferring resistance to gentamicin and apramycin in strains of *Salmonella typhimurium* phage type 204c isolated in Britain.. J Hyg (Lond).

[49] Kado CI, Liu ST (1981). Rapid procedure for detection and isolation of large and small plasmids.. J Bacteriol.

[50] Datsenko KA, Wanner BL (2000). One-step inactivation of chromosomal genes in *Escherichia coli* K-12 using PCR products.. Proc Natl Acad Sci U S A.

[51] Struve C, Bojer M, Krogfelt KA (2008). Characterization of *Klebsiella pneumoniae* type 1 fimbriae by detection of phase variation during colonization and infection and impact on virulence.. Infect Immun.

[52] Chaveroche MK, Ghigo JM, d'Enfert C (2000). A rapid method for efficient gene replacement in the filamentous fungus *Aspergillus nidulans*.. Nucleic Acids Res.

[53] Reisner A, Molin S, Zechner EL (2002). Recombinogenic engineering of conjugative plasmids with fluorescent marker cassettes.. FEMS Microbiol Ecol.

[54] Altschul SF, Madden TL, Schäffer AA, Zhang J, Zhang Z (1997). Gapped BLAST and PSI-BLAST: a new generation of protein database search programs.. Nucleic Acids Res.

[55] Larkin MA, Blackshields G, Brown NP, Chenna R, McGettigan PA (2007). Clustal W and Clustal X version 2.0.. Bioinformatics.

